# Increasing Hospitalizations for Wernicke Encephalopathy in Spain: A Nationwide Population-Based Study

**DOI:** 10.3390/jcm15041549

**Published:** 2026-02-15

**Authors:** David Puertas-Miranda, M. A. Ortiz-Pinto, F. Josue Cordero-Pérez, Luis Arribas-Pérez, P. Martinez-Rodríguez, Antonio-J. Chamorro, Miguel Marcos

**Affiliations:** 1 Department of Internal Medicine, University Hospital of Salamanca, 37007 Salamanca, Spain; davidpuertasmiranda@gmail.com (D.P.-M.); 1amartinezpablo@gmail.com (P.M.-R.); ajchamorro@usal.es (A.-J.C.); 2 Institute of Biomedical Research of Salamanca (IBSAL), 37007 Salamanca, Spain; josue@usal.es (F.J.C.-P.); larribas@saludcastillayleon.es (L.A.-P.); 3 Department of Medicine, University of Salamanca, 37007 Salamanca, Spain; 4 Department of Internal Medicine, Complejo Asistencial de Ávila, 05071 Ávila, Spain; mairaalejadra@gmail.com; 5 Department of Internal Medicine, Complejo Asistencial de Zamora, 49022 Zamora, Spain

**Keywords:** Wernicke encephalopathy, epidemiology, hospital mortality, hospital discharge data, health care economics

## Abstract

**Background/Objectives:** Wernicke encephalopathy (WE) is an acute neurological syndrome caused by severe thiamine deficiency. Early detection is challenging due to the low sensitivity of the classic triad. **Methods:** This retrospective observational study used the Spanish Minimum Basic Data Set, including hospital admissions with a primary diagnosis of WE (2016–2022). Demographic, clinical, and economic variables were also analyzed. Severity of illness (SOI) and risk of mortality (ROM) were assessed using the All Patient Refined Diagnosis-Related Groups (APR–DRG) system. **Results:** A total of 2477 WE episodes were included (1864 men; mean age, 58.2 years; standard deviation [SD], 11.0). The hospital admission rate increased by an average of 16% per year (incidence rate ratio [IRR], 1.16; *p* < 0.001). The proportion of foreign-born patients increased significantly over the study period. Most patients were discharged home (1868; 75.4%), whereas transfers to residential care facilities increased over time. The mean hospital stay was 19.0 days (SD 36.5). In-hospital mortality was 3.7%. In multivariable analysis, malnutrition (odds ratio [OR] 1.64), cancer (OR 2.11), and active infection (OR 5.79) were independently associated with mortality. The incorporation of ROM into the mortality model markedly improved discrimination, and mortality increased progressively with higher ROM categories: moderate (OR 3.45), major (OR 11.76), and extreme (OR 38.76) (all *p* < 0.001). **Conclusions:** WE is an increasingly frequent cause of neurological hospitalization in Spain, associated with a substantial clinical and economic burden. In-hospital mortality is driven mainly by overall clinical complexity and comorbidity burden rather than by WE in isolation.

## 1. Introduction

Wernicke encephalopathy (WE) is an acute neurological syndrome caused by severe thiamine (vitamin B1) deficiency [1,2]. If left untreated, it may progress to the chronic stage of the Wernicke–Korsakoff spectrum, characterized by persistent amnesia and confabulation (Korsakoff’s psychosis) [3,4]. First described by Carl Wernicke in 1881, the condition has been traditionally associated with a triad of confusion, ophthalmoplegia, and ataxia. However, this triad is observed in only a minority of patients, hindering early recognition [5,6,7,8]. Given the low sensitivity of this triad (10–38%) [9,10], Caine et al. [8] proposed four diagnostic criteria: dietary deficiency, oculomotor abnormalities, cerebellar dysfunction, and either altered mental state or mild memory impairment. The presence of at least two of these criteria yields nearly 100% sensitivity among patients with chronic alcohol use without hepatic encephalopathy [8]. Current European guidelines recommend applying these criteria in alcohol-related settings and other at-risk populations. Nonetheless, WE remains underdiagnosed due to its variable clinical presentation and the low level of suspicion outside alcohol-related contexts [11,12,13,14].

Traditionally, WE was considered almost exclusive to individuals with chronic excessive alcohol consumption, since ethanol interferes with thiamine absorption and metabolism [4,15]. It is now recognized across a wider range of conditions, including malnutrition, bariatric surgery, anorexia nervosa, malignancy, and prolonged hypermetabolic states, where timely vitamin B1 supplementation is essential to prevent irreversible complications [9,16,17]. Over the past decade, demographic changes, such as population aging and the increasing frequency of metabolic surgery, have modified risk profiles, creating new scenarios of subclinical thiamine deficiency in frail, multimorbid patients or those with prolonged intensive care stays. These changes highlight the need to assess epidemiologic patterns and develop preventive protocols adapted to current healthcare settings [18,19,20,21,22].

Available evidence on WE is mostly limited to hospital-based series or selected cohorts, which restricts their generalizability and precludes assessment of regional and temporal trends [23]. To date, only a limited number of nationwide studies have explored the epidemiology of WE in Europe, highlighting the scarcity of population-based data in this field [24,25]. Although we have previously examined the clinical and epidemiological profile of WE in Spain in a multicenter study [26,27,28], such studies were not designed to capture nationwide trends or to comprehensively assess healthcare utilization and outcomes over time. In this context, nationwide administrative databases, such as the Minimum Basic Data Set (MBDS) [29], provide a valuable opportunity to address these gaps by enabling large-scale analyses of temporal trends, geographic variation, and factors associated with clinical severity, in-hospital mortality, and healthcare costs. To our knowledge, this study represents the first national-level analysis of the epidemiological, clinical, and economic burden of WE in Spain. The aim of this study was to analyze temporal trends, geographic distribution, and factors associated with clinical severity, in-hospital mortality, and healthcare costs of WE in Spain, in order to inform strategies for prevention, early detection, and management.

## 2. Materials and Methods

### 2.1. Study Design and Inclusion Criteria

This retrospective cohort study used nationwide population data from the MBDS, which records hospital admissions across Spain. The study period was from 2016 to 2022. We included all hospitalizations with a primary or secondary diagnosis of WE, coded as E51.2 according to the International Classification of Diseases, Tenth Revision (ICD-10) [30]. For the main analysis, only episodes with WE as the primary diagnosis were considered to ensure that the hospitalization was directly attributable to the disease. Admissions with missing information on key variables (age, sex, discharge outcome, or region of origin) were excluded to ensure data quality.

### 2.2. Data Sources and Variables

We obtained the data by submitting a formal request to the Ministry of Health, in compliance with the national and European Union data protection regulations. The MBDS includes anonymized information on more than 95% of all hospital admissions in Spain. Each record contains clinical, demographic, administrative, and economic variables, with up to 20 secondary diagnoses and procedures coded per admission using the ICD-10.

The dataset provided information on the length of hospital stay and discharge destination (home, community health center, transfer to another hospital, voluntary discharge, or death). We used the All Patient Refined Diagnosis-Related Groups (APR–DRG) system to assess the clinical complexity and in-hospital mortality risk. This classification, widely applied in the MBDS, integrates data on the main diagnosis, secondary diagnoses (comorbidities and complications), age, procedures performed, and other clinical and demographic variables. It generates two indicators: severity of illness (SOI) and risk of mortality (ROM). Both were categorized into four levels (minor, moderate, major, or extreme). The SOI reflects the overall clinical severity based on comorbidities (e.g., cirrhosis, renal failure, and sepsis) and complex interventions, such as major surgery or mechanical ventilation. ROM estimates the probability of in-hospital death using predictive models derived from large databases. Each admission is automatically classified into one of the four SOI and ROM levels, allowing standardized comparisons across patients with different diagnoses and procedures.

Administrative and economic data included the total hospitalization costs expressed in euros (EUR) and adjusted annually. Costs were derived from the MBDS cost-weighting system. Crude hospital admission and mortality rates of WE were calculated using annual population estimates from the Spanish National Institute of Statistics (INE, by its initials in Spanish) [31].

Additional variables included the patient’s country of birth, categorized as Spanish-born or foreign-born, temporal distribution of admissions, crude rates by year and sex, and geographic distribution across Spanish regions. We also assessed the admission type by distinguishing between scheduled and emergency admissions.

The primary outcome was in-hospital mortality, selected as a clinically relevant and unambiguous endpoint that is reliably captured in administrative hospital databases. Secondary outcomes included length of hospital stay, discharge destination, type of admission (scheduled or emergency), and hospitalization costs. Temporal trends in hospital admission rates, mortality, SOI, ROM, and costs were also assessed.

### 2.3. Statistical Analysis

Categorical variables were described as absolute frequencies and percentages, and continuous variables as mean values with standard deviations or, when not normally distributed, as median values with interquartile ranges. Hospital admission rates of WE were calculated per 100,000 population, and mortality rates per 1,000,000 population, using official population estimates from the INE.

Qualitative variables were compared using the chi-squared test or Fisher’s exact test when the expected frequencies were <5. Temporal trends in hospital admission and mortality rates were assessed using Poisson regression models, with year as the independent variable and population size as the offset, estimating annual rate ratios (IRRs) with 95% confidence intervals (CIs).

Continuous variables were analyzed using Student’s t-test for normally distributed data, and the Mann–Whitney or Kruskal–Wallis tests otherwise. Factors associated with in-hospital mortality were first explored in univariate analyses, including sociodemographic, clinical, and health care variables. Variables with *p* < 0.20 in the univariate analyses and clinically relevant covariates were considered as candidates for multivariable models. In these multivariable logistic regression models, the ROM index was included as a categorical variable with four levels (minor, moderate, major, and extreme), using the lowest risk category as the reference group, in order to avoid imposing linear trend assumptions. We then fitted multivariable logistic regression models and compared them using likelihood ratio tests (for nested models), changes in the Akaike Information Criterion (AIC), the area under the ROC curve (AUC), and model parsimony.

Model performance was evaluated using the minus 2 Log Likelihood (−2LL), Nagelkerke’s R^2^, Hosmer–Lemeshow goodness-of-fit test, AIC, and AUC with 95% confidence intervals. Statistical significance was set at *p* < 0.05. All analyses were performed using Stata v.17 (StataCorp, College Station, TX, USA) and SPSS v.28.0.1.1 (IBM Corp., Armonk, NY, USA).

## 3. Results

The Results are organized into four main sections addressing overall burden and temporal trends, regional and demographic patterns, clinical outcomes and hospital resource utilization, and factors associated with in-hospital mortality.

### 3.1. Overall Burden and Temporal Trends

Between 2016 and 2022, there were 2477 hospital admissions in Spain with WE as the main diagnosis in the MBDS database. Of these, 1864 (75.2%) were men, with an overall mean age of 58.2 years (SD 11.0).

During the study period, the crude hospital admission rate for WE increased by 128.9%, from 0.45 per 100,000 inhabitants (n = 211) in 2016 to 1.03 per 100,000 (n = 495) in 2022 (Table 1). Trend analysis showed an average annual increase of 16% in WE hospital admission rates (IRR per year = 1.16; 95% CI 1.14–1.18; *p* < 0.001).

In sex- and year-stratified analyses, men consistently showed a slightly higher mean age than that of women. The mean age remained stable among men (approximately 59 years), whereas a progressive increase was observed among women, from 54.4 years in 2016 to 56.5 years in 2022 (Table 2).

Hospital admission rates showed marked regional variability across Spain, with higher values observed in Catalonia, the Valencian Community, and Galicia (Supplementary Table S1), as illustrated by the geographical distribution of crude morbidity rates shown in Figures S1–S7 for the period 2016–2022.

Of all admissions, 1756 patients (70.9%) were Spanish-born, and 721 (29.1%) were foreign-born. The proportion of foreign-born patients increased significantly over the study period, from 56 (23.9%) in 2017 to 151 (31.0%) in 2021 (*p* < 0.001) (Supplementary Table S2).

### 3.2. Clinical Outcomes and Hospital Length of Stay

Most patients were discharged home (1868; 75.4%). However, this proportion declined over the study period, from 177 (83.9%) in 2016 to 367 (74.1%) in 2022. In parallel, transfers to residential care facilities increased substantially, from 6 admissions (2.8%) in 2016 to 55 (11.1%) in 2022 (Table 3).

Hospital admission rates are expressed per 100,000 inhabitants, and mortality rates per 1,000,000 inhabitants.

**Figure 1:**
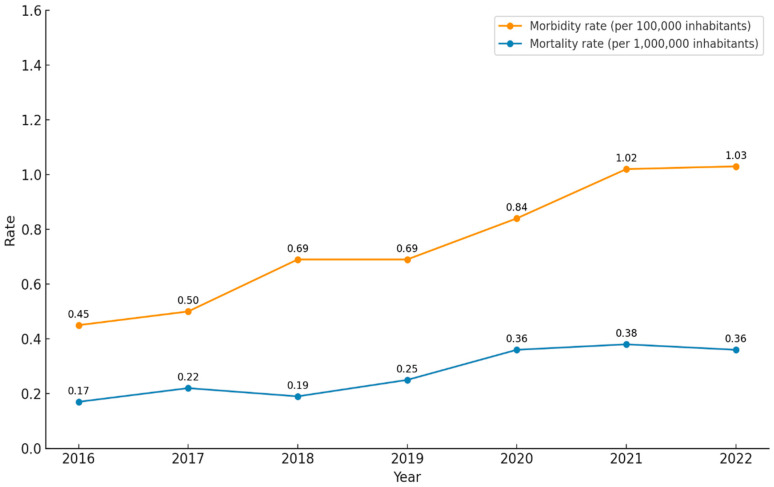
Trends in hospital admission rates and population mortality rates for Wernicke encephalopathy in Spain (2016–2022).

Overall, the in-hospital mortality rate was 3.7% (n = 91) and remained relatively stable throughout the study period. In contrast, crude population mortality rates attributable to WE showed a significant upward trend, with an average relative annual increase of 15% (IRR per year = 1.15; 95% CI 1.04–1.28; *p* = 0.008). Despite this trend, the absolute number of deaths remained low, ranging from 8 to 18 deaths per year. Figure 1 illustrates the annual evolution of WE admission and crude population mortality rates during the study period.

Other discharge destinations included transfer to another hospital (155, 6.3%) and voluntary discharge (51, 2.1%), with no relevant temporal variation (Supplementary Table S3).

The mean hospital length of stay was 19.0 days (SD 36.5), with no significant overall differences between the sexes. However, marked interannual variability was observed. In 2017 and 2019, statistically significant sex-related differences were identified, with longer hospital stays among women, whose mean length of stay was 23.1 days (SD 24.1) compared with 16.8 days (SD 18.2) in men in 2017 (*p* < 0.005), and 18.7 days (SD 19.7) compared with 13.8 days (SD 13.5) in 2019 (*p* < 0.005). Detailed annual and sex-specific distributions of length of stay are provided in Supplementary Table S4.

The distribution of urgent and scheduled admissions during the study period is shown in Supplementary Table S5.

### 3.3. APR–DRG Classification System Variables and Cost per Episode

The APR–DRG SOI and ROM indicators showed a wide distribution of hospital admissions. For SOI, the most frequent category was moderate (1183, 47.8%), followed by major (658, 26.6%) and extreme (159, 6.4%); no significant sex differences were observed (Supplementary Table S6). Over time, the proportion of major admissions rose slightly (from 25.6% in 2016 to 27.5% in 2022), and the proportion of patients with extreme severity increased from 3.3% to 5.9%, although these changes were not statistically significant (Supplementary Table S7). For ROM, more than half of the patients were classified as having minor risk (1295; 52.3%), followed by moderate risk (756; 30.5%), major risk (317; 12.8%), and extreme risk (109; 4.4%). Men showed a slightly higher proportion of major risk (13.2% vs. 11.6% in women); however, this difference was not statistically significant (Supplementary Table S8). Temporal analysis revealed a significant decrease in minor risk admissions (from 63.0% in 2016 to 50.9% in 2022; *p* = 0.005) and an increase in major risk (from 10.4% to 15.2%; *p* = 0.004), and extreme risk also rose (from 3.3% in 2016 to 5.1% in 2022), although the difference was not statistically significant (Supplementary Table S9).

The mean cost per hospital admission for WE during the study period was EUR 5362 (95% CI 5150–5574), with no clinically relevant differences by sex. The estimated total hospitalization cost attributable to WE between 2016 and 2022 was EUR 13.08 million, with a progressive increase in annual expenditure parallel to the increase in the number of admissions (Supplementary Tables S10 and S11 and Supplementary Figure S8).

### 3.4. Factors Associated with Mortality

Clinical and severity-related factors associated with in-hospital mortality were analyzed using univariate and multivariable logistic regression analyses. The univariate analysis included age, sex, alcohol use, and the ten main comorbidities recorded in the database (alcoholic liver disease, diabetes mellitus, atrial fibrillation, obesity, heart failure, malnutrition, gastrointestinal surgery, cancer, active infection on admission, and psychiatric disorders), together with the APR–DRG severity indicators (SOI, and ROM; Supplementary Table S12).

Among the comorbidities evaluated, malnutrition, observed in 537 patients (22.5%) among survivors and 30 (33.0%) among non-survivors, was significantly associated with higher in-hospital mortality (*p* = 0.020). Similarly, cancer was present in 206 (8.6%) of survivors and 15 (16.5%) of non-survivors (*p* = 0.010), and active infection on admission was recorded in 111 (4.7%) of survivors and 20 (22.0%) of non-survivors (*p* < 0.001).

Mortality increased progressively with higher SOI and ROM categories (*p* < 0.001 for both), reaching the highest values in the groups classified as having extreme severity (SOI: 37.4% vs. 5.2% in the minor severity category) and extreme risk of mortality (ROM: 33.0% vs. 3.3% in the minor risk category). Given the strong correlation between the two indices (Pearson r = 0.715; Spearman ρ = 0.681; *p* < 0.001 for both tests), only the ROM index was included in the multivariable models to avoid collinearity (Supplementary Table S13).

In the multivariable analysis (Table 4), Model 1 included clinical comorbidities, Model 2 included the ROM index and comorbidities, and Model 3 included ROM alone. In Model 1, malnutrition (OR 1.64; 95% CI 1.04–2.58; *p* = 0.034), cancer (OR 2.11; 95% CI 1.18–3.79; *p* = 0.012), and active infection on admission (OR 5.79; 95% CI 3.39–9.89; *p* < 0.001) were independently associated with in-hospital mortality.

After including the ROM index (Model 2), the explanatory power of the model increased substantially (Nagelkerke R^2^ = 0.216 vs. 0.062 in Model 1; AUC = 0.826 vs. 0.641, respectively). Using the minor ROM category as the reference, the adjusted odds of death rose progressively with higher ROM categories: moderate (OR 3.45; 95% CI 1.61–7.40; *p* = 0.001), major (OR 11.76; 95% CI 5.58–24.79; *p* < 0.001), and extreme (OR 38.76; 95% CI 17.58–85.48; *p* < 0.001). The overall effect of ROM on mortality was significant (*p* < 0.001), showing a consistent upward trend across risk categories. In this model, infection remained borderline significant (OR 1.80; 95% CI 0.98–3.30; *p* = 0.056), whereas cancer and malnutrition were not statistically significant. Model 3, which included only ROM, showed a similar discriminative performance (AUC = 0.823) and good calibration (Hosmer–Lemeshow test *p* = 1.000). No collinearity was detected among the independent variables (all VIF < 1.2). The ROC curves corresponding to the three models are shown in Supplementary Figures S9–S11.

## 4. Discussion

This study, based on one of the largest nationwide cohorts of Wernicke encephalopathy published to date, provides an updated perspective on its epidemiology in Spain and demonstrates a statistically significant increase in hospital admissions over time. Crude hospital admission rates doubled, with an average relative annual increase of approximately 16%. These findings confirm the growing clinical relevance of Wernicke encephalopathy in hospital settings [32,33,34].

This increase may reflect, at least in part, improved identification of WE in clinical practice, possibly related to greater awareness and the wider availability of neuroimaging techniques, such as magnetic resonance imaging. Nevertheless, a true increase in hospital admissions cannot be excluded, particularly in the context of persistent alcohol use disorders, nutritional deficiencies, and increasingly complex comorbidity profiles in aging populations [11,23,35].

The marked predominance of men observed in our cohort is consistent with previous national studies and with population-based registry data from other European countries, reinforcing the consistency of the epidemiological profile of WE across hospital settings in Europe [36,37,38]. The proportion of male patients in our series closely mirrors that reported in the only two studies based on nationwide hospital registries published to date: Palm et al. in Finland (2022) [24], in which men accounted for 73.2% of cases, and Rasiah et al. in Switzerland (2019) [25], with 70.8% male patients. Compared with previous national data [26], the relative proportion of women appears to have increased, suggesting a gradual shift away from the classical demographic profile of this disease [11].

A relevant finding was the progressive increase in hospital admissions among foreign-born patients, which rose from 23.9% of cases in 2017 to 31.0% in 2021. This increase clearly exceeded the proportional growth of the foreign-born population residing in Spain over the same period, which rose from 9.8% to 11.5%, according to official registries [31]. This discrepancy suggests that, beyond demographic changes, factors such as nutritional vulnerability, socioeconomic conditions, and barriers to early diagnosis may contribute to a higher risk of WE in these populations [5,26,39,40].

From a clinical perspective, most patients were discharged home; however, the proportion of transfers to community or long-term care facilities increased during the study period. This finding likely reflects greater clinical complexity and a growing burden of comorbidities among patients with WE, reinforcing the notion that this condition increasingly affects patients with more complex clinical profiles, departing from the classical phenotype historically described for this disease [27,41].

The economic burden associated with WE is considerable. The mean cost per admission was comparable to the national average for hospital discharges in Spain; however, the prolonged length of hospital stay translated into substantial cumulative expenditure, exceeding EUR 13 million over the study period. These results highlight the potential economic benefits of the early identification and prevention of thiamine deficiency in high-risk populations [42,43].

Analysis of the APR–DRG classification system variables confirmed the broad clinical spectrum of WE, with a relevant proportion of patients classified into higher SOI and ROM categories and a significant temporal shift toward higher ROM categories [26,44]. In-hospital mortality remained relatively stable, with a mean rate of 3.7%, which was lower than that reported in previous national cohorts. This reduction may reflect improvements in the early diagnosis and timely administration of thiamine [27,45].

In the multivariable analysis, malnutrition, cancer, and active infection were associated with an increased risk of in-hospital mortality. However, after adjusting for overall clinical complexity using the ROM index, this variable emerged as the main determinant of mortality, showing a strong and progressive association across increasing risk categories. These findings underscore that, beyond etiological treatment with thiamine, the clinical course of WE largely depends on early identification and management of coexisting conditions, whose correction may substantially influence in-hospital outcomes. Although the ROM index is derived from administrative data and has limited direct clinical applicability, its robust statistical performance supports its use as an adjustment variable and illustrates the cumulative prognostic impact of the comorbidity burden in population-based analyses, reinforcing its utility in this population-based context rather than for individual-level prognostic prediction [22,46,47,48,49,50,51,52,53].

## 5. Limitations

The main strength of this study lies in its large sample size and nationwide scope based on the Spanish MBDS, which provides near-universal coverage of hospital admissions. Nevertheless, several limitations should be acknowledged.

The reliance on ICD-10 diagnostic coding may lead to underestimation of cases, particularly in mild or atypical presentations of WE, as administrative databases tend to preferentially capture hospitalizations with more severe or classical manifestations. This limitation should be considered when interpreting the observed temporal trends, particularly in non-alcohol-related contexts, which have traditionally been less well recognized in administrative records. In this regard, restricting the main analysis to hospitalizations in which WE was recorded as the primary diagnosis, while ensuring that admissions were directly attributable to the disease, may have further contributed to an underestimation of the overall disease burden, particularly in patients admitted for comorbid conditions in whom WE was coded as a secondary diagnosis.

In addition, information on some comorbidities associated with malnutrition and neurological complications (e.g., HIV infection) may be incompletely captured in administrative hospital databases, limiting a more detailed assessment of their potential contribution in this cohort. Likewise, cost estimates derived from administrative hospital data primarily reflect direct inpatient costs and do not capture indirect costs or post-discharge healthcare utilization, which may result in an underestimation of the overall economic burden associated with WE.

Finally, the retrospective observational design of the study precludes causal inference. Despite these limitations, the consistency of our findings with those of previous population-based studies supports their validity.

## 6. Conclusions

In conclusion, this study demonstrates that WE continues to represent a relevant neurological challenge in the hospital setting, with increasing hospital admission rates, substantial healthcare resource utilization, and mortality primarily determined by overall clinical complexity and comorbidity burden. In addition to early thiamine administration, prompt identification and management of associated conditions, such as malnutrition, cancer, and infection, are essential to improve prognosis. From a preventive and clinical perspective, these findings highlight the importance of early identification of nutritional risk, maintaining a low threshold for empirical thiamine supplementation in well-recognized high-risk populations, and systematically considering Wernicke encephalopathy in the presence of acute neurological symptoms in these risk groups. Given the low cost and favorable safety profile of thiamine, such preventive and early intervention strategies may represent a cost-effective approach to reducing the clinical and economic burden of the disease. This study provides a solid framework to guide future research and healthcare planning to prevent thiamine deficiency and optimize the management of patients at risk of WE.

## Figures and Tables

**Table 1:** Frequency and crude annual hospital admission rates for Wernicke encephalopathy in Spain (2016–2022).

Year	N	Percentage (%)	95% CI	Annual Crude Rate ^1^	Annual Crude Rate in Adults (>18 Years) ^2^
2016	211	8.5	7.5–9.7	0.45	0.56
2017	234	9.4	8.4–10.7	0.50	0.62
2018	325	13.1	11.8–14.5	0.69	0.85
2019	327	13.2	11.9–14.6	0.69	0.85
2020	398	16.1	14.6–17.6	0.84	1.03
2021	487	19.7	18.1–21.2	1.02	1.25
2022	495	20.0	18.4–21.6	1.03	1.27

N = number of hospital admissions. ^1^ Crude rate per 100,000 inhabitants, based on the mid-year population (July 1st of the corresponding year), according to data from the Spanish National Institute of Statistics (INE). ^2^ Crude rate per 100,000 inhabitants, based on the mid-year population (July 1st of the corresponding year), excluding individuals under 18 years old, according to data from the INE.

**Table 2:** Mean age of Wernicke encephalopathy admissions by year and sex in Spain (2016–2022).

Year	N	Total (n = 2477)		Male (n = 1864)		Female (n = 613)		*p*-Value
		Mean (SD)	95% CI	Mean (SD)	95% CI	Mean (SD)	95% CI	
2016	211	57.8 (11.4)	56.2–59.3	59.3 (11.0)	57.5–61.0	54.4 (11.7)	51.5–57.3	0.0043
2017	234	58.3 (11.1)	56.9–59.7	58.7 (10.5)	57.1–60.3	57.2 (12.6)	54.0–60.4	0.4035
2018	325	58.4 (11.3)	57.2–59.6	59.2 (10.7)	57.8–60.6	56.1 (12.7)	53.3–58.9	0.0483
2019	327	58.1 (10.7)	57.0–59.3	58.2 (10.7)	56.9–59.5	57.8 (11.0)	55.0–60.6	0.7536
2020	398	58.2 (10.5)	57.1–59.2	58.6 (10.2)	57.4–59.7	56.8 (11.5)	54.4–59.2	0.1499
2021	487	57.9 (11.0)	56.9–58.9	58.9 (10.4)	57.8–60.0	54.8 (12.1)	52.6–57.0	0.0012
2022	495	58.5 (10.2)	57.5–59.4	59.1 (9.5)	58.2–60.1	56.5 (11.9)	54.4–58.6	0.0242

N = number of hospital admissions. Data are expressed as mean values with standard deviations (SDs) and 95% confidence intervals (CIs). An independent samples t-test was used to compare the mean age between sexes. For 2018, 2021, and 2022, Welch’s t-test (assuming unequal variances) was applied.

**Table 3:** Distribution of hospital discharge types among Wernicke encephalopathy admissions by discharge year in Spain (2016–2022).

Type of Discharge	2016 (n = 211)		2017 (n = 234)		2018 (n = 325)		2019 (n = 327)		2020 (n = 398)		2021 (n = 487)		2022 (n = 495)	
	n (%)	95% CI	n (%)	95% CI	n (%)	95% CI	n (%)	95% CI	n (%)	95% CI	n (%)	95% CI	n (%)	95% CI
Home	177 (83.9)	78.2–88.5	186 (79.5)	73.7–84.4	238 (73.2)	68.0–77.9	239 (73.1)	67.9–77.8	291 (73.1)	68.4–77.4	370 (76.0)	71.9–79.7	367 (74.1)	70.0–77.9
Transfer to a residential care facility	6 (2.8)	1.0–6.0	14 (6.0)	3.9–10.8	35 (10.8)	7.6–14.6	37 (11.3)	8.0–15.2	46 (11.6)	8.5–15.1	40 (8.2)	5.9–11.0	55 (11.1)	8.4–14.2
Transfer to another hospital	16 (7.6)	4.4–12.0	14 (6.0)	3.9 –10.8	25 (7.7)	5.0–11.1	17 (5.2)	3.0–8.1	24 (6.0)	3.9–8.8	30 (6.2)	4.1–8.6	29 (5.9)	3.9–8.3
Death	8 (3.8)	1.6–7.3	10 (4.3)	2.0–7.7	9 (2.8)	1.2–5.1	12 (3.7)	1.9–6.3	17 (4.3)	2.5–6.7	18 (3.7)	2.2–5.7	17 (3.4)	2.0–5.4
Other	1 (0.5)	0.0–2.6	3 (1.3)	0.2–3.7	6 (1.9)	0.6–3.9	10 (3.1)	1.4–5.5	14 (3.5)	1.9–5.8	20 (4.1)	2.5–6.2	16 (3.2)	1.8–5.1
Voluntary discharge	3 (1.4)	0.3–4.0	6 (2.6)	0.9–5.4	10 (3.1)	1.4–5.5	10 (3.1)	1.4–5.5	5 (1.3)	0.4–2.9	8 (1.6)	0.7–3.2	9 (1.8)	0.8–3.4
No information	–	–	1 (0.4)	0.0–2.3	2 (0.6)	0.0–2.2	2 (0.6)	0.6–3.9	1 (0.2)	0.0–1.3	1 (0.2)	0.0–1.1	2 (0.4)	0.0–1.4

N = number of hospital admissions.

**Table 4:** Multivariable logistic regression models for predicting in-hospital mortality.

Variables	Model 1: Clinical Comorbidities	Model 2: Comorbidities and ROM	Model 3: ROM Only
Malnutrition	1.64 (1.04–2.58); *p* = 0.034	1.05 (0.65–1.69); *p* = 0.846	—
Cancer	2.11 (1.18–3.79); *p* = 0.012	1.57 (0.86–2.90); *p* = 0.145	—
Infection	5.79 (3.39–9.89); *p* < 0.001	1.80 (0.98–3.30); *p* = 0.056	—
ROM Moderate *	—	3.45 (1.61–7.40); *p* = 0.001	3.67 (1.72–7.84); *p* < 0.001
ROM Major *	—	11.76 (5.58–24.79); *p* < 0.001	13.43 (6.49–27.79); *p* < 0.001
ROM Extreme *	—	38.76 (17.58–85.48); *p* < 0.001	48.80 (23.03–103.39); *p* < 0.001
–2 Log Likelihood	738.43	630.75	635.91
Nagelkerke R^2^	0.062	0.216	0.209
Hosmer–Lemeshow χ^2^	1.21 (*p* = 0.547)	5.08 (*p* = 0.407)	0.00 (*p* = 1.000)
AIC	746.4	644.8	643.9
AUC	0.641	0.826	0.823

Data are expressed as odds ratios (ORs) with 95% confidence intervals (CIs). Model 1 includes clinical comorbidities; Model 2 adds the APR–DRG Risk of Mortality (ROM) index; Model 3 includes ROM only. Model fit was assessed using the Hosmer–Lemeshow test, and discriminative ability was assessed using the area under the ROC curve (AUC). AIC = Akaike Information Criterion. Multicollinearity was evaluated using the Variance Inflation Factor (VIF); no relevant collinearity was detected (all VIF < 1.2 in Models 1 and 2). * Compared with ROM Minor as the reference category.

## Data Availability

The data presented in this study are contained within the article and the Supplementary Materials.

## Supplementary Materials

The following supporting information can be downloaded at: https://www.mdpi.com/article/10.3390/jcm15041549/s1. Figure S1: Geographical distribution of the crude morbidity rate of Wernicke encephalopathy in Spain, 2016; Figure S2: Geographical distribution of the crude morbidity rate of Wernicke encephalopathy in Spain, 2017; Figure S3: Geographical distribution of the crude morbidity rate of Wernicke encephalopathy in Spain, 2018; Figure S4: Geographical distribution of the crude morbidity rate of Wernicke encephalopathy in Spain, 2019; Figure S5: Geographical distribution of the crude morbidity rate of Wernicke encephalopathy in Spain, 2020; Figure S6: Geographical distribution of the crude morbidity rate of Wernicke encephalopathy in Spain, 2021; Figure S7: Geographical distribution of the crude morbidity rate of Wernicke encephalopathy in Spain, 2022; Figure S8: Distribution of hospital costs for Wernicke encephalopathy in Spain (2016–2022); Figure S9: ROC curve for Model 1 (clinical comorbidities); Figure S10: ROC curve for Model 2 (clinical comorbidities and risk of mortality); Figure S11: ROC curve for Model 3 (risk of mortality only); Table S1: Distribution of Wernicke encephalopathy admissions and crude rates by autonomous community in Spain (2016–2022); Table S2: Annual distribution of Wernicke encephalopathy admissions by nationality (Spanish vs. foreign-born), 2016–2022; Table S3: Overall distribution of hospital discharge types in Wernicke encephalopathy admissions in Spain (2016–2022); Table S4: Mean length of hospital stay for Wernicke encephalopathy by sex in Spain (2016–2022); Table S5: Distribution of urgent and scheduled admissions in Wernicke encephalopathy cases in Spain (2016–2022); Table S6: Distribution of clinical severity according to the All Patient Refined Diagnosis Related Groups classification in Wernicke encephalopathy admissions in Spain (2016–2022); Table S7: Temporal distribution of clinical severity according to the All Patient Refined Diagnosis Related Groups classification in Wernicke encephalopathy admissions in Spain (2016–2022); Table S8: Distribution of risk of in-hospital mortality according to the All Patient Refined Diagnosis Related Groups classification in Wernicke encephalopathy admissions in Spain (2016–2022); Table S9: Temporal distribution of risk of in-hospital mortality according to the All Patient Refined Diagnosis Related Groups classification in Wernicke encephalopathy admissions in Spain (2016–2022); Table S10: Distribution of healthcare costs associated with Wernicke encephalopathy in Spain (2016–2022); Table S11: Estimated total annual hospitalization costs for Wernicke encephalopathy in Spain (2016–2022); Table S12: Baseline clinical characteristics and comorbidities of hospitalized patients according to survival status (2016–2022); Table S13: Correlation between the Severity of Illness (SOI) and Risk of Mortality (ROM) indices.

## References

[1] Harper C., Fornes P., Duyckaerts C., Lecomte D., Hauw J.J. (1995). An international perspective on the prevalence of the Wernicke–Korsakoff syndrome. Metab. Brain Dis..

[2] Galvin R., Bråthen G., Ivashynka A., Hillbom M., Tanasescu R., Leone M.A. (2010). EFNS guidelines for diagnosis, therapy and prevention of Wernicke encephalopathy. Eur. J. Neurol..

[3] Victor M., Adams R.D., Collins G.H. (1989). The Wernicke–Korsakoff Syndrome and Related Neurologic Disorders due to Alcoholism and Malnutrition.

[4] Miller K.L., Trifan G., Testai F.D. (2019). Neurology of nutritional deficiencies. Curr. Neurol. Neurosci. Rep..

[5] Sechi G., Serra A. (2007). Wernicke’s encephalopathy: New clinical settings and recent advances in diagnosis and management. Lancet Neurol..

[6] Kopelman M.D., Thomson A.D., Guerrini I., Marshall E.J. (2009). The Korsakoff syndrome: Clinical aspects, psychology and treatment. Alcohol. Alcohol..

[7] Infante M.T., Fancellu R., Murialdo A., Barletta L., Castellan L., Serrati C. (2016). Challenges in diagnosis and treatment of Wernicke encephalopathy: Report of 2 cases. Nutr. Clin. Pract..

[8] Caine D., Halliday G.M., Kril J.J., Harper C.G. (1997). Operational criteria for the classification of chronic alcoholics: Identification of Wernicke’s encephalopathy. J. Neurol. Neurosurg. Psychiatry.

[9] Sinha S., Kataria A., Kolla B.P., Thusius N., Loukianova L.L. (2019). Wernicke encephalopathy—Clinical pearls. Mayo Clin. Proc..

[10] Zuccoli G., Gallucci M., Capellades J., Regnicolo L., Tumiati B., Giadás T.C., Bottari W., Mandrioli J., Bertolini M. (2007). Wernicke encephalopathy: MR findings at clinical presentation in twenty-six alcoholic and nonalcoholic patients. AJNR Am. J. Neuroradiol..

[11] Puertas-Miranda D., Díaz-Ávila E.G., Llamas-Alonso C., Novo-Veleiro I., Chamorro A.J., Marcos M. (2025). Alcohol-related and non-alcohol-related Wernicke encephalopathy: A systematic review and meta-analysis of epidemiology and clinical features. Neuroepidemiology.

[12] Oudman E., Wijnia J.W., Oey M., van Dam M., Painter R.C., Postma A. (2019). Wernicke’s encephalopathy in hyperemesis gravidarum: A systematic review. Eur. J. Obstet. Gynecol. Reprod. Biol..

[13] Chandrakumar A., Bhardwaj A., ‘t Jong G.W. (2019). Review of thiamine deficiency disorders: Wernicke encephalopathy and Korsakoff psychosis. J. Basic. Clin. Physiol. Pharmacol..

[14] Cantu-Weinstein A., Branning R., Alamir M., Weleff J., Do M., Nero N., Anand A. (2024). Diagnosis and treatment of Wernicke’s encephalopathy: A systematic literature review. Gen. Hosp. Psychiatry.

[15] Bond N.W., Homewood J. (1991). Wernicke’s encephalopathy and Korsakoff’s psychosis: To fortify or not to fortify?. Neurotoxicol. Teratol..

[16] Ahmad S., Ikram S., Dunn B.K. (2022). Fatal Wernicke’s encephalopathy with cardiovascular involvement in a young psychiatric patient. Am. J. Med. Sci..

[17] Oudman E., Wijnia J.W., Oey M.J., van Dam M., Postma A. (2021). Wernicke’s encephalopathy in Crohn’s disease and ulcerative colitis. Nutrition.

[18] Yu A.T., Gross A., Park K., Harvey E.J. (2023). Wernicke encephalopathy after bariatric surgery: A literature review. Obes. Surg..

[19] Cobilinschi C., Andrei C.A., Grințescu I.M., Mirea L. (2024). Metabolic failure due to thiamine deficiency during critical illness. Curr. Opin. Clin. Nutr. Metab. Care.

[20] Gomes F., Bergeron G., Bourassa M.W., Fischer P.R. (2021). Thiamine deficiency unrelated to alcohol consumption in high-income countries: A literature review. Ann. N. Y. Acad. Sci..

[21] Chen C.C., Chang P.C., Chang T.W., Chuang H.Y. (2024). Wernicke encephalopathy after Roux-en-Y gastric bypass presenting with altered mental status: A video case report. Obes. Surg..

[22] Wijnia J.W., Oudman E., Bresser E.L., Gerridzen I.J., van de Wiel A., Beuman C., Mulder C.L. (2014). Need for early diagnosis of mental and mobility changes in Wernicke encephalopathy. Cogn. Behav. Neurol..

[23] Isenberg-Grzeda E., Kutner H.E., Nicolson S.E. (2012). Wernicke–Korsakoff syndrome: Under-recognized and under-treated. Psychosomatics.

[24] Palm A., Vataja R., Talaslahti T., Ginters M., Kautiainen H., Elonheimo H., Suvisaari J., Lindberg N., Koponen H. (2022). Incidence and mortality of alcohol-related dementia and Wernicke–Korsakoff syndrome: A nationwide register study. Int. J. Geriatr. Psychiatry.

[25] Rasiah R., Gregoriano C., Mueller B., Kutz A., Schuetz P. (2024). Hospital outcomes in medical patients with alcohol-related and non-alcohol-related Wernicke encephalopathy. Mayo Clin. Proc..

[26] Chamorro A.J., Rosón-Hernández B., Medina-García J.A., Muga-Bustamante R., Fernández-Solá J., Martín-González M.-C., Seco-Hernández E., Novo-Veleiro I., Suárez-Cuervo C., Mateos-Díaz A.M. (2017). Differences between alcoholic and nonalcoholic patients with Wernicke encephalopathy: A multicenter observational study. Mayo Clin. Proc..

[27] Novo-Veleiro I., Mateos-Díaz A.M., Rosón-Hernández B., Medina-García J.A., Muga R., Fernández-Solá J., Martín-González M.-C., Seco-Hernández E., Suárez-Cuervo C., Monte-Secades R. (2023). Treatment variability and its relationships to outcomes among patients with Wernicke’s encephalopathy: A multicenter retrospective study. Drug Alcohol. Depend..

[28] Novo-Veleiro I., Herrera-Flores J., Rosón-Hernández B., Medina-García J.A., Muga R., Fernández-Solá J., Martín-González M.-C., Seco-Hernández E., Suárez-Cuervo C., Mateos-Díaz A.-M. (2022). Alcoholic liver disease among patients with Wernicke encephalopathy: A multicenter observational study. Drug Alcohol. Depend..

[29] Spanish Ministry of Health Conjunto Mínimo Básico de Datos (CMBD). Madrid: Government of Spain. https://www.sanidad.gob.es/estadEstudios/estadisticas/cmbd.htm.

[30] World Health Organization (1992). International Statistical Classification of Diseases and Related Health Problems: 10th Revision (ICD-10).

[31] National Statistics Institute (INE) Population by Year, Sex and Age Group. National Results. Madrid: National Statistics Institute. https://www.ine.es/jaxiT3/Tabla.htm?t=31304.

[32] Wijnia J.W. (2022). A clinician’s view of Wernicke–Korsakoff syndrome. J. Clin. Med..

[33] Harper C. (1979). Wernicke’s encephalopathy: A more common disease than realised. A neuropathological study of 51 cases. J. Neurol. Neurosurg. Psychiatry.

[34] Kohnke S., Meek C.L. (2021). Don’t seek, don’t find: The diagnostic challenge of Wernicke’s encephalopathy. Ann. Clin. Biochem..

[35] Gascón-Bayarri J., Campdelacreu J., García-Carreira M.C., Estela J., Martínez-Yélamos S., Palasí A., Delgado T., Reñé R. (2011). Wernicke encephalopathy in non-alcoholic patients: A series of 8 cases. Neurología.

[36] Elipe Miravet M., Cervigón Carrasco V., Fernández García O., Estruch V., Ballester Arnal R. (2021). Excessive alcohol consumption: Are there gender differences?. Rev. INFAD Psicol..

[37] Bríñez Horta J.A. (2001). Gender differences in alcohol-related problems according to consumption level. Adicciones.

[38] Victor M., Yakovlev P.I.S.S. (1955). Korsakoff’s psychic disorder in conjunction with peripheral neuritis: A translation of Korsakoff’s original article with comments on the author and his contribution to clinical medicine. Neurology.

[39] Gonzalez-Quintela A., Fernandez-Conde S., Alves M.-T., Campos J., Lopez-Raton M., Puerta R., Monte R., Gude F. (2011). Temporal and spatial patterns in the rate of alcohol withdrawal syndrome in a defined community. Alcohol.

[40] Smith T.J., Johnson C.R., Koshy R., Hess S.Y., Qureshi U.A., Mynak M.L., Fischer P.R. (2021). Thiamined eficiency disorders: A clinical perspective. Ann. N. Y. Acad. Sci..

[41] Scalzo S.J., Bowden S.C., Ambrose M.L., Whelan G., Cook M.J. (2015). Wernicke–Korsakoff syndrome not related to alcohol use: A systematic review. J. Neurol. Neurosurg. Psychiatry.

[42] Spanish Ministry of Health (2022). Methodological Note on Hospital Costs. Minimum Basic Data Set (MBDS).

[43] Sicras-Mainar A., Velasco-Velasco S. (2007). Resource utilization and costs in patients seeking care for neurological disorders in primary care. Rev. Neurol..

[44] Jophlin L., Liu T.Y., McClain C.J. (2024). Nutritional deficiencies in alcohol use disorder/alcohol-associated liver disease. Curr. Opin. Gastroenterol..

[45] Mateos-Díaz A.M., Marcos M., Chamorro A.J. (2022). Wernicke–Korsakoff syndrome and other diseases associated with thiamine deficiency. Med. Clin..

[46] McCormick P.J., Lin H.M., Deiner S.G., Levin M.A. (2018). Validation of the All Patient Refined Diagnosis Related Group (APR-DRG) Risk of Mortality and Severity of Illness Modifiers as a measure of perioperative risk. J. Med. Syst..

[47] Elgwairi E., Yang S., Nugent K. (2021). Association of the All-Patient Refined Diagnosis-Related Groups Severity of Illness and Risk of Mortality classification with outcomes. South. Med. J..

[48] Santos J.V., Viana J., Pinto C., Souza J., Lopes F., Freitas A., Lopes S. (2022). All Patient Refined–Diagnosis Related Groups’ (APR–DRGs) Severity of Illness and Risk of Mortality as predictors of in-hospital mortality. J. Med. Syst..

[49] Paro M.R., Ramanan S., McNeill I.T., Bulsara K.R. (2023). Predictive validity of the All Patients Refined Diagnosis Related Group modifiers for costs and outcomes from intracranial hemorrhage. J. Neurosurg..

[50] Mifsud F., Messager D., Jannot A.S., Védie B., Balanant N.A., Poghosyan T., Flamarion E., Carette C., Lucas-Martini L., Czernichow S. (2022). Clinical diagnosis, outcomes and treatment of thiamine deficiency in a tertiary hospital. Clin. Nutr..

[51] Latt N., Dore G. (2014). Thiamine in the treatment of Wernicke encephalopathy in patients with alcohol use disorders. Intern. Med. J..

[52] Wallis W.E., Willoughby E., Baker P. (1978). Coma in the Wernicke–Korsakoff syndrome. Lancet.

[53] Donnino M.W., Vega J., Miller J., Walsh M. (2007). Myths and misconceptions of Wernicke’s encephalopathy: What every emergency physician should know. Ann. Emerg. Med..

