# Supplementary material for: Increasing Hospitalizations for Wernicke Encephalopathy in Spain: A Nationwide Population-Based Study
Source: J Clin Med. 2026 Feb 15;15(4):1549. doi: 10.3390/jcm15041549 (PMC12941529; doi:10.3390/jcm15041549)
Supplement: Supplementary file 1 [file jcm-15-01549-s001.zip › jcm-4119786-supplementary.pdf]

Supplementary material

Supplementary Table S1. Distribution of Wernicke encephalopathy admissions and crude rates by autonomous community in Spain (2016–2022).

Autonomous Community	Total[1]	2016		2017		2018		2019		2020		2021		2022	
		N cases	Crude rate[2] (per 100,000)	N cases	Crude rate[2] (per 100,000)	N cases	Crude rate[2] (per 100,000)	N cases	Crude rate[2] (per 100,000)	N cases	Crude rate[2] (per 100,000)	N cases	Crude rate[2] (per 100,000)	N cases	Crude rate[2] (per 100,000)
Andalusia	320	41	0.49	39	0.46	40	0.48	35	0.41	56	0.66	59	0.69	50	0.59
Aragon	46	6	0.46	8	0.61	3	0.23	5	0.38	8	0.60	11	0.84	5	0.38
Principality of Asturias	28	3	0.29	3	0.29	6	0.59	3	0.29	7	0.69		0.00	5	0.50
Balearic Islands	62	1	0.09	12	1.04	10	0.85	14	1.17	8	0.66	4	0.33	13	1.05
Canary Islands	96	9	0.42	8	0.37	23	1.05	14	0.63	11	0.49	18	0.80	13	0.57
Cantabria	21	1	0.17	3	0.52	1	0.17	4	0.69	4	0.69	5	0.86	3	0.51
Castile and Leon	85	4	0.16	8	0.33	12	0.50	8	0.33	15	0.63	16	0.67	22	0.93
Castile-La Mancha	94	6	0.29	13	0.64	10	0.49	10	0.49	20	0.98	16	0.78	19	0.92
Catalonia	506	0	0.00	0	0.00	65	0.86	86	1.13	99	1.29	129	1.68	127	1.65
Valencian Community	313	37	0.75	41	0.83	37	0.75	31	0.62	44	0.87	51	1.01	72	1.41
Extremadura	40	3	0.28	3	0.28	3	0.28	7	0.66	11	1.04	8	0.76	5	0.48
Galicia	151	17	0.63	17	0.63	16	0.59	19	0.70	22	0.82	38	1.41	22	0.82
Madrid, Community of	459	59	0.92	59	0.91	67	1.02	55	0.82	56	0.83	82	1.22	81	1.19
Murcia, Region of	81	4	0.27	6	0.41	9	0.61	15	1.00	16	1.06	18	1.19	13	0.85
Navarra, Chartered Community of	17	1	0.16	0	0.00	3	0.46	1	0.15	1	0.15	4	0.61	7	1.06
Basque Country	106	15	0.69	8	0.37	12	0.55	12	0.55	13	0.59	21	0.96	25	1.15
Rioja, La	15	1	0.32	1	0.32	4	1.28	3	0.95	1	0.32	2	0.63	3	0.95
Ceuta	2	0	0.00	0	0.00	1	1.18	1	1.18	0	0.00	0	0.00	0	0.00
Melilla	1	0	0.00	1	1.18	0	0.00	0	0.00	0	0.00	0	0.00	0	0.00

N = number of hospital admissions.

[1] Totals exclude 34 admissions corresponding to patients residing abroad.

[2] Crude rates per 100,000 inhabitants were calculated using mid-year population estimates from the Spanish National Institute of Statistics (INE, by its initials in Spanish).

For each autonomous community, annual values are presented as absolute numbers of admissions and corresponding crude rates. Years with no recorded admissions are shown as 0 cases and a crude rate of 0.00.

Supplementary Table S2. Annual distribution of Wernicke encephalopathy admissions by nationality (Spanish vs. foreign–born), 2016–2022.

Year	N	Spanish (n=1756)		Foreign-born (n=721)		p-value[1]
		N (%)	95% CI	N (%)	95% CI	
2016	211	120 (56.9)	49.9 – 63.6	91 (43.1)	36.3 – 50.1	0.0470
2017	234	178 (76.1)	70.1 – 81.3	56 (23.9)	18.6 – 29.9	<0.001
2018	325	241 (74.2)	69.0 – 78.8	84 (25.9)	21.1 – 30.9	<0.001
2019	327	239 (73.1)	67.9 – 77.8	88 (26.9)	22.1 – 32.0	<0.001
2020	398	295 (74.1)	69.5 – 78.3	103 (25.9)	21.6 – 30.4	<0.001
2021	487	336 (69.0)	64.6 – 73.0	151 (31.0)	26.9 – 35.3	<0.001
2022	495	347 (70.1)	65.8 – 74.1	148 (29.9)	25.8 – 34.1	<0.001

N = number of hospital admissions.
Data are expressed as absolute numbers (n) and percentages (%), with 95% confidence intervals (CI). Percentages were calculated using the total number of admissions per year as the denominator.
[1] Two-sample test for equality of proportions.

Supplementary Table S3. Overall distribution of hospital discharge types in Wernicke encephalopathy admissions in Spain (2016–2022).

Type of discharge	N	Percentage (%)	95% CI
Home	1868	75.4	73.6 – 77.0
Transfer to a residential care facility	233	9.4	8.0 – 10.6
Transfer to another hospital	155	6.3	5.4 – 7.2
Death	91	3.7	2.9 – 4.4
Other	70	2.8	2.2 – 3.5
Voluntary discharge	51	2.1	1.5 – 2.6
Missing information	9	0.4	0.1 – 0.6

N = number of hospital admissions.
Data are expressed as absolute numbers (n) and percentages (%), with 95% confidence intervals (CI).

Supplementary Table S4. Mean length of hospital stay for Wernicke encephalopathy by sex in Spain (2016–2022).

Length of hospital stay, days	Total		Male		Female		p-value[1]
	Mean (SD)	95% CI	Mean (SD)	95% CI	Mean (SD)	95% CI	
Total	19.0 (36.5)	17.5 – 20.4	18.6 (39.5)	16.8 – 20.4	20.0 (25.0)	18.0 – 22.0	0.425
2016	17.6 (19.9)	14.9 – 20.3	16.3 (16.2)	13.7 – 19.0	20.5 (26.6)	13.9 – 27.2	0.158
2017	18.5 (20.2)	15.9 – 21.1	16.8 (18.2)	14.0 – 19.5	23.1 (24.4)	16.9 – 29.3	0.033
2018	24.2 (69.5)	16.6 – 31.8	26.8 (79.9)	16.7 – 37.0	16.6 (18.2)	12.7 – 20.6	0.249
2019	14.7 (15.0)	13.1 – 16.3	13.8 (13.5)	12.1 – 15.4	18.7 (19.7)	13.7 – 23.6	0.019
2020	19.2 (27.6)	16.5 – 21.9	17.8 (23.0)	15.2 – 20.4	23.7 (39.0)	15.6 – 31.8	0.072
2021	17.9 (27.6)	15.4 – 20.4	17.7 (29.6)	14.6 – 20.7	18.5 (20.4)	14.9 – 22.2	0.765
2022	20.1 (39.9)	16.6 – 23.6	20.2 (44.5)	15.7 – 24.8	19.8 (21.7)	15.9 – 23.6	0.909

Data are expressed as mean values with standard deviations (SD) and 95% confidence intervals (CI).
[1] p-value derived from independent samples t-test comparing mean length of stay between sexes.

Supplementary Table S5. Distribution of urgent and scheduled admissions in Wernicke encephalopathy cases in Spain (2016–2022).

	Urgent (n=2345)		Scheduled (n=128)	
**Year**	**N (%)**	**95% CI**	**N (%)**	**95% CI**
2016	197 (93.4)	89.1 – 96.3	13 (6.2)	3.3 – 10.3
2017	215 (91.9)	87.6 – 95.0	19 (8.1)	4.9 – 12.3
2018	305 (93.9)	90.6 – 96.2	18 (5.5)	3.1 – 8.0
2019	318 (97.3)	94.8 – 98.7	8 (2.5)	1.0 – 4.7
2020	373 (93.7)	90.8 – 95.8	25 (6.3)	4.1 – 9.1
2021	468 (96.1)	93.9 – 97.6	19 (3.9)	2.3 – 6.0
2022	469 (94.7)	92.3 – 96.5	26 (5.2)	3.4 – 7.6

N = number of hospital admissions.
Data are expressed as absolute numbers (n) and percentages (%), with 95% confidence intervals (CI).
Four records were excluded due to missing data (one in 2016, two in 2018, and one in 2019).

Supplementary Table S6. Distribution of clinical severity according to the All Patient Refined Diagnosis Related Groups classification in Wernicke encephalopathy admissions in Spain (2016–2022).

Severity level	Total		Male		Female		p-value[1]
	**N (%)**	**95% CI**	**N (%)**	**95% CI**	**N (%)**	**95% CI**	
Minor	477 (19.3)	17.7 – 20.8	345 (18.5)	16.7 – 20.3	132 (21.5)	18.3 – 25.0	0.396
Moderate	1183 (47.8)	45.7 – 49.7	903 (48.4)	46.1 – 50.7	280 (45.7)	41.6 – 49.7	
Major	658 (26.6)	24.8 – 28.3	497 (26.7)	24.6 – 28.7	161 (26.3)	22.8 – 29.9	
Extreme	159 (6.4)	5.4 – 7.4	119 (6.4)	5.3 – 7.5	40 (6.5)	4.7 – 8.7	

N = number of hospital admissions.
[1]Chi-squared test comparing severity level and sex.

Supplementary Table S7. Temporal distribution of clinical severity according to the All Patient Refined Diagnosis Related Groups classification in Wernicke encephalopathy admissions in Spain (2016–2022).

Severity of illness	2016 N (%)	2017 N (%)	2018 N (%)	2019 N (%)	2020 N (%)	2021 N (%)	2022 N (%)	p-value[1]
Minor	50 (23.7)	38 (16.2)	63 (19.4)	62 (19.0)	80 (20.1)	85 (17.5)	99 (20.0)	0.488
Moderate	100 (47.4)	122 (52.1)	162 (49.8)	176 (53.8)	176 (44.2)	216 (44.4)	231 (46.7)	0.069
Major	54 (25.6)	58 (24.8)	76 (23.4)	73 (22.3)	114 (28.6)	147 (30.2)	136 (27.5)	0.145
Extreme	7 (3.3)	16 (6.8)	24 (7.4)	16 (4.9)	28 (7.0)	39 (8.0)	29 (5.9)	0.255

N = number of hospital admissions.
[1] p-value from chi-square test across years. The linear-by-linear association (trend test) was also non-significant (p = 0.641 for minor, p = 0.064 for moderate, p = 0.053 for major, and p = 0.303 for extreme).

Supplementary Table S8. Distribution of risk of in-hospital mortality according to the All Patient Refined Diagnosis Related Groups classification in Wernicke encephalopathy admissions in Spain (2016–2022).

Mortality risk	Total		Male		Female		p-value[1]
	N (%)	95% CI	N (%)	95% CI	N (%)	95% CI	
Low	1295 (52.3)	50.2 – 54.2	960 (51.5)	49.2 – 53.7	335 (54.7)	50.6 – 58.6	0.382
Moderate	756 (30.5)	28.7 – 32.3	571 (30.6)	28.5 – 32.7	185 (30.2)	26.5 – 33.9	
High	317 (12.8)	11.5 – 14.1	246 (13.2)	11.6 – 14.8	71 (11.6)	9.1 – 14.3	
Extreme	109 (4.4)	3.6 – 5.5	87 (4.7)	3.7 – 5.7	22 (3.6)	2.2 – 5.3	

N = number of hospital admissions.
[1] Chi-square test comparing mortality risk levels by sex.

Supplementary Table S9. Temporal distribution of risk of in-hospital mortality according to the All Patient Refined Diagnosis Related Groups classification in Wernicke encephalopathy admissions in Spain (2016–2022).

Risk of mortality	2016 N (%)	2017 N (%)	2018 N (%)	2019 N (%)	2020 N (%)	2021 N (%)	2022 N (%)	p-value[1]
Minor	133 (63.0)	123 (52.6)	172 (52.9)	175 (53.5)	205 (51.5)	235 (48.3)	252 (50.9)	0.034
Moderate	49 (23.2)	68 (29.1)	104 (32.0)	107 (32.7)	129 (32.4)	156 (32.0)	143 (28.9)	0.208
Major	22 (10.4)	28 (12.0)	32 (9.8)	31 (9.5)	53 (13.3)	76 (15.6)	75 (15.2)	0.044
Extreme	7 (3.3)	15 (6.4)	17 (5.2)	14 (4.3)	11 (2.8)	20 (4.1)	25 (5.1)	0.369

N = number of hospital admissions.
[1] Chi-square test across years. Linear-by-linear association (trend test) indicated a significant upward trend for minor (p = 0.005) and major (p = 0.004) categories, while no significant trends were observed for moderate (p = 0.293) or extreme (p = 0.800).

Supplementary Table S10. Distribution of healthcare costs associated with Wernicke encephalopathy in Spain (2016–2022).

Mean hospitalization cost (EUR)	Total		Male		Female		p-value[1]
	Mean	95% CI	Mean	95% CI	Mean	95% CI	
**Total**	5,362.0	5,130.4 – 5,593.6	5,326.1	5,070.2 – 5,581.9	5,134.5	4,779.1 – 5,490.0	0.443
2016	4,263.5	3,924.8 – 4,592.6	4,256.7	3,856.5 – 4,656.8	4,263.5	3,640.9 – 4,886.0	0.985
2017	5,019.7	4,257.4 – 5,782.1	5,073.2	4,098.8 – 6,047.6	4,871.4	3,846.3 – 5,896.5	0.818
2018	5,905.6	4,946.1 – 6,865.0	6,256.2	4,985.5 – 7,526.9	4,899.6	4,199.5 – 5,599.6	0.223
2019	5,010.0	4,317.7 – 5,702.4	4,957.2	4,246.7 – 5,667.7	5,231.4	3,168.5 – 7,294.2	0.759
2020	5,273.4	4,912.9 – 5,633.8	5,117.4	4,851.6 – 5,383.1	5,792.3	4,493.8 – 7,090.7	0.120
2021	5,590.8	5,223.2 – 5,958.4	5,627	5,177.1 – 6,076.8	5,481.3	4,890.9 – 6,071.8	0.737
2022	5,298.9	4,907.7 – 5,690.2	5,401.3	4,899.8 – 5,902.7	5,002.5	4,533.6 – 5,471.3	0.382

[1] p-value derived from independent samples t-test comparing mean hospitalization costs between sexes.

Supplementary Table S11. Estimated total annual hospitalization costs for Wernicke encephalopathy in Spain (2016–2022).

Year	N	Mean cost per admission (EUR)	Estimated total annual cost (EUR)
2016	211	4,263.5	899,628
2017	234	5,019.7	1,173,610
2018	325	5,905.6	1,919,320
2019	327	5,010.0	1,638,270
2020	398	5,273.4	2,099,800
2021	487	5,590.8	2,722,769
2022	495	5,298.9	2,623,456
Total	2,477	5,278.6	13,077,000

Annual costs were estimated by multiplying the number of admissions by the mean cost per admission derived from the Minimum Basic Data Set (MBDS).
N = number of hospital admissions.

Supplementary Table S12. Baseline clinical characteristics and comorbidities of hospitalized patients according to survival status (2016–2022).

**Variable**		**Survivor (n = 2386) N (%)**	**Non-survivors (n = 91) N (%)**	**OR (95% CI)**	**p-value**
Sex (male)		1797 (75.3)	67 (73.6)	1.10 (0.68 – 1.76)	0.714
Age (years, mean, SD)		58.1 (10.9)	60.7 (9.6)	–	0.025
Alcoholic liver disease		293 (12.3)	10 (11.0)	0.88 (0.45 – 1.72)	0.712
Diabetes mellitus		353 (14.8)	15 (16.5)	1.14 (0.65 – 2.00)	0.657
Atrial fibrillation		109 (4.6)	4 (4.4)	0.96 (0.35 – 2.67)	0.938
Obesity		113 (4.7)	3 (3.3)	0.69 (0.21 – 2.20)	0.524
Heart failure		137 (5.7)	3 (3.3)	0.56 (0.18 – 1.79)	0.322
Malnutrition		537 (22.5)	30 (33.0)	1.69 (1.08 – 2.65)	0.020
Gastrointestinal surgery		89 (3.7)	1 (1.1)	2.87 (0.40 – 20.8)	0.188
Cancer		206 (8.6)	15 (16.5)	2.09 (1.18 – 3.70)	0.010
Infection		111 (4.7)	20 (22.0)	5.77 (3.39 – 9.82)	<0.001
Psychiatric disorder		199 (8.3)	5 (5.5)	0.64 (0.26 – 1.59)	0.332
Severity of Illness (SOI)	Minor	474 (19.9)	3 (3.3)	0.14 (0.04–0.44)	<0.001
	Moderate	1167 (48.9)	16 (17.6)	0.22 (0.13–0.39)	<0.001
	Major	620 (26.0)	38 (41.8)	2.04 (1.33–3.13)	<0.001
	Extreme	125 (5.2)	34 (37.4)	10.79 (6.90–17.11)	<0.001
Risk of Mortality (ROM)	Minor	1285 (53.9)	10 (11.0)	0.11 (0.06–0.21)	<0.001
	Moderate	735 (30.8)	21 (23.1)	0.67 (0.41–1.11)	0.116
	Major	287 (12.0)	30 (33.0)	3.60 (2.28–5.66)	<0.001
	Extreme	79 (3.3)	30 (33.0)	14.36 (8.79–23.47)	<0.001

Comparisons were performed using the chi-squared test or Student’s t-test, as appropriate. Odds ratios (OR) and 95% confidence intervals (CI) were calculated using univariate logistic regression.
N = number of hospital admissions.

Supplementary Table S13. Correlation between the Severity of Illness (SOI) and Risk of Mortality (ROM) indices.

Type of correlation	Correlation coefficient (r / ρ)	p-value	N
Pearson's *r*	0.715	<0.001	2,477
Spearman's ρ	0.681	<0.001	2,477

Pearson's r was used for parametric correlation, and Spearman's ρ for rank-based correlation. Both tests showed a strong positive association ($p < 0.001$).

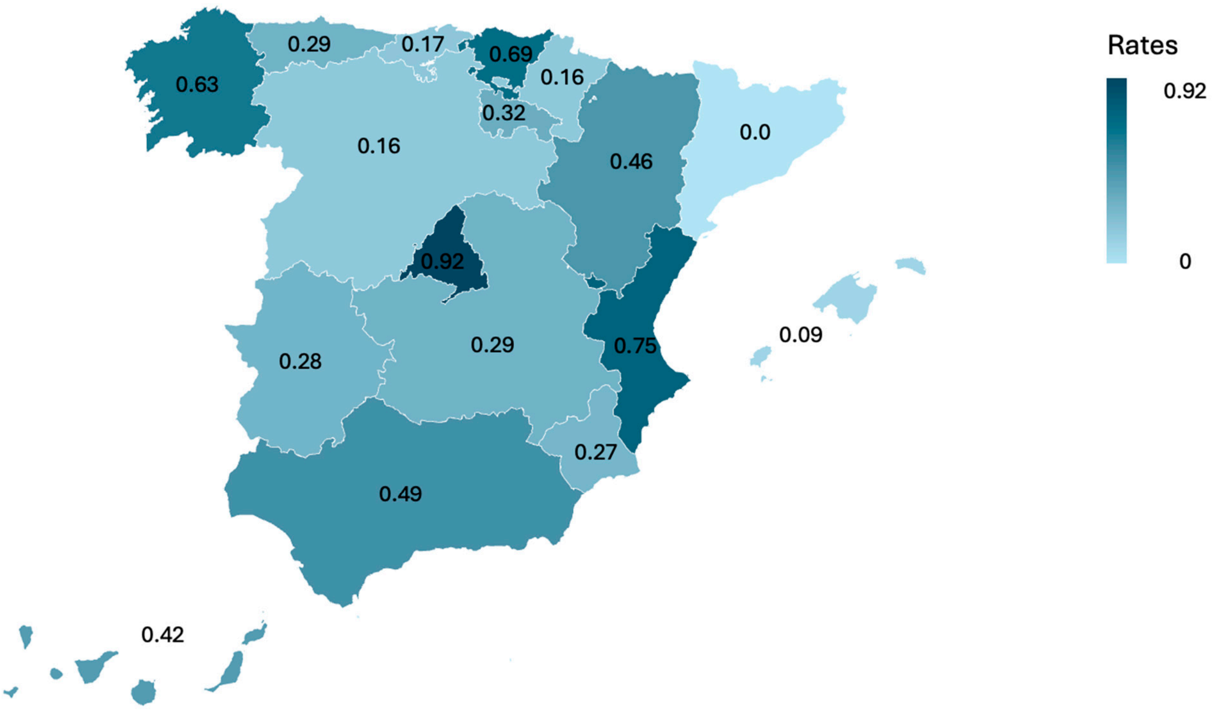

Supplementary Figure S1. Geographical distribution of the crude morbidity rate of Wernicke encephalopathy in Spain, 2016.

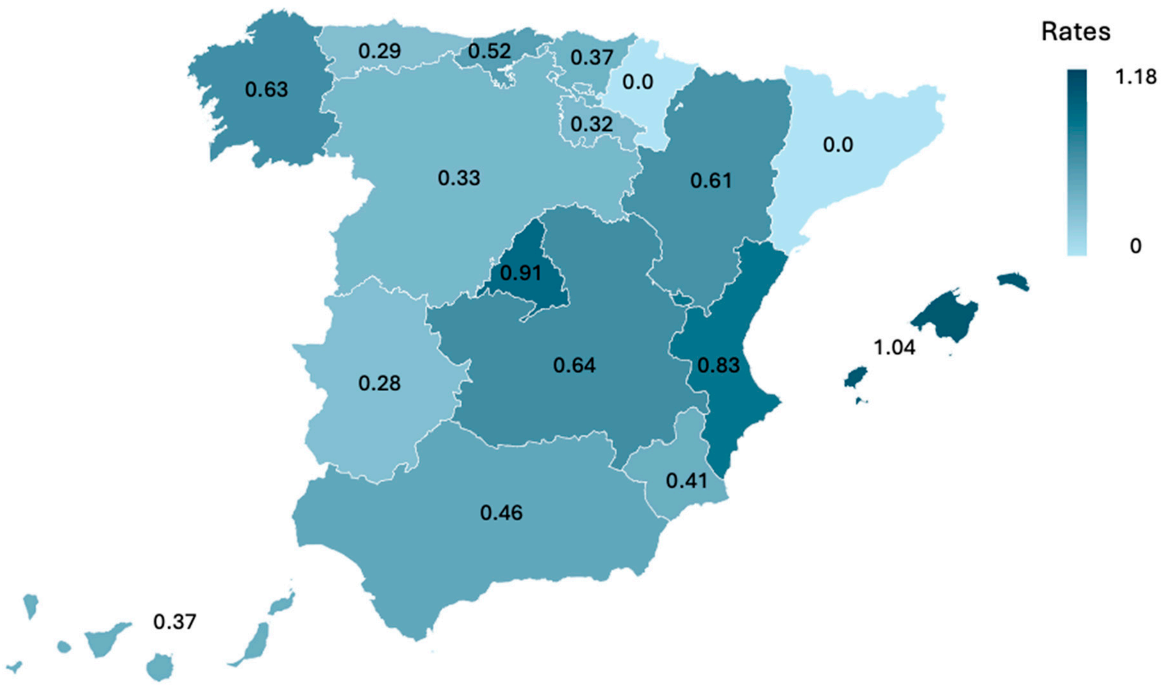

Supplementary Figure S2. Geographical distribution of the crude morbidity rate of Wernicke encephalopathy in Spain, 2017.

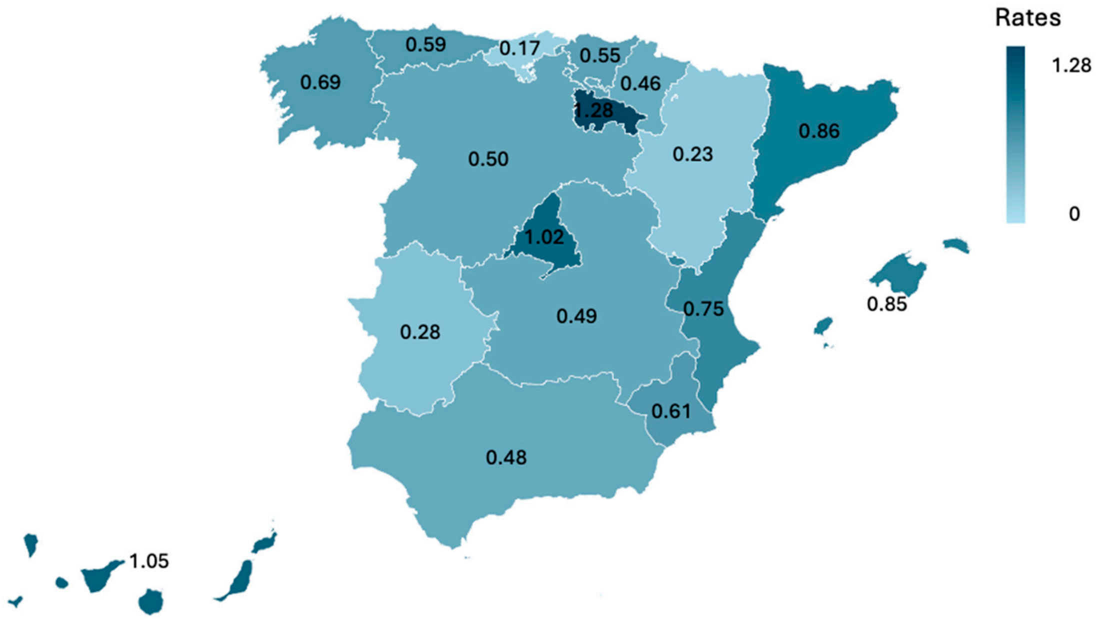

Supplementary Figure S3. Geographical distribution of the crude morbidity rate of Wernicke encephalopathy in Spain, 2018.

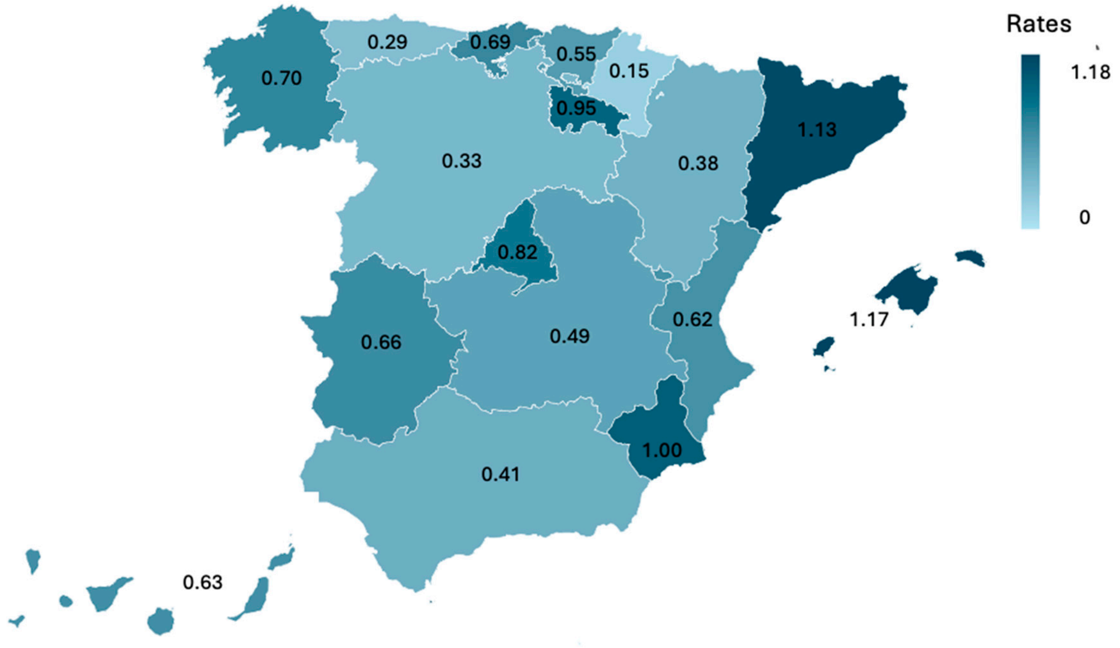

Supplementary Figure S4. Geographical distribution of the crude morbidity rate of Wernicke encephalopathy in Spain, 2019.

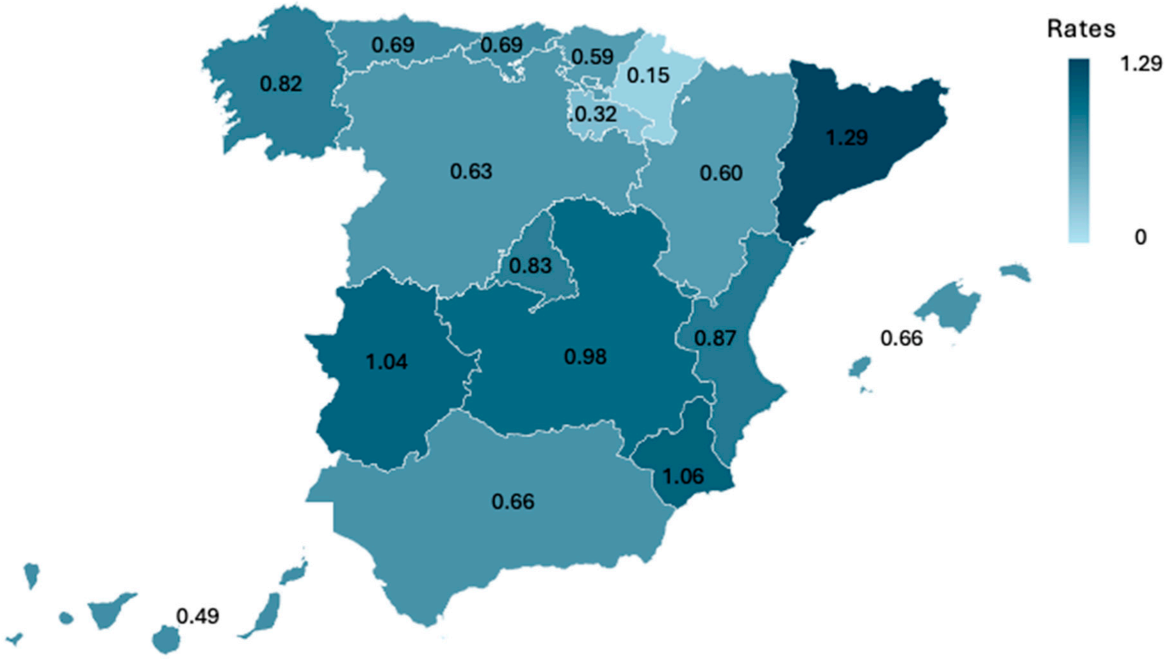

Supplementary Figure S5. Geographical distribution of the crude morbidity rate of Wernicke encephalopathy in Spain, 2020.

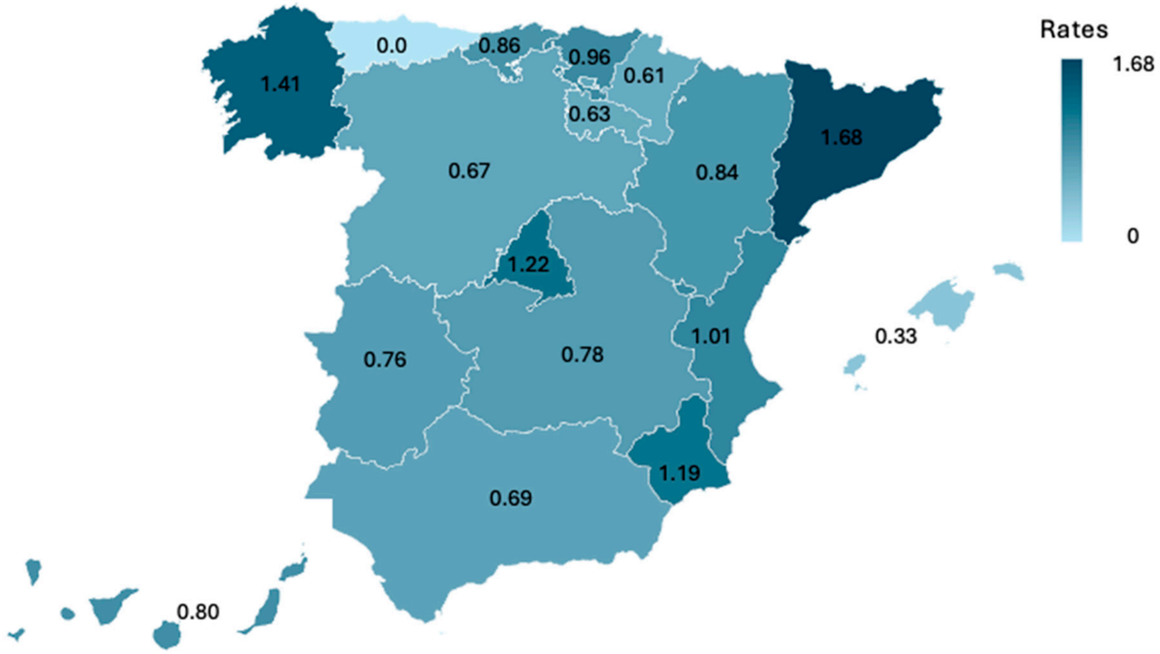

Supplementary Figure S6. Geographical distribution of the crude morbidity rate of Wernicke encephalopathy in Spain, 2021.

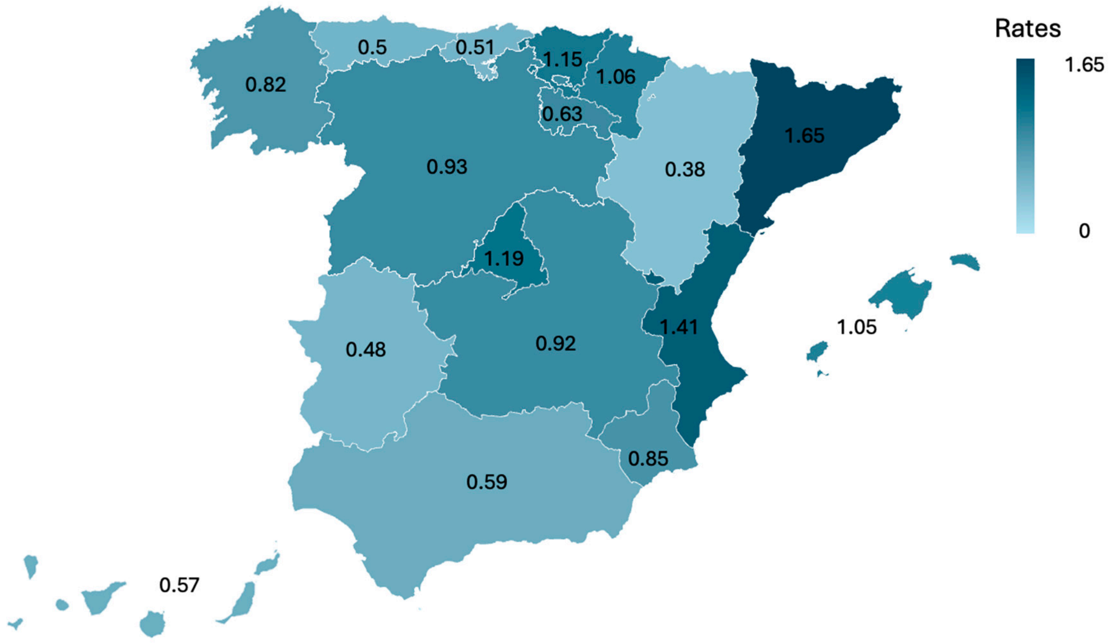

Supplementary Figure S7. Geographical distribution of the crude morbidity rate of Wernicke encephalopathy in Spain, 2022.

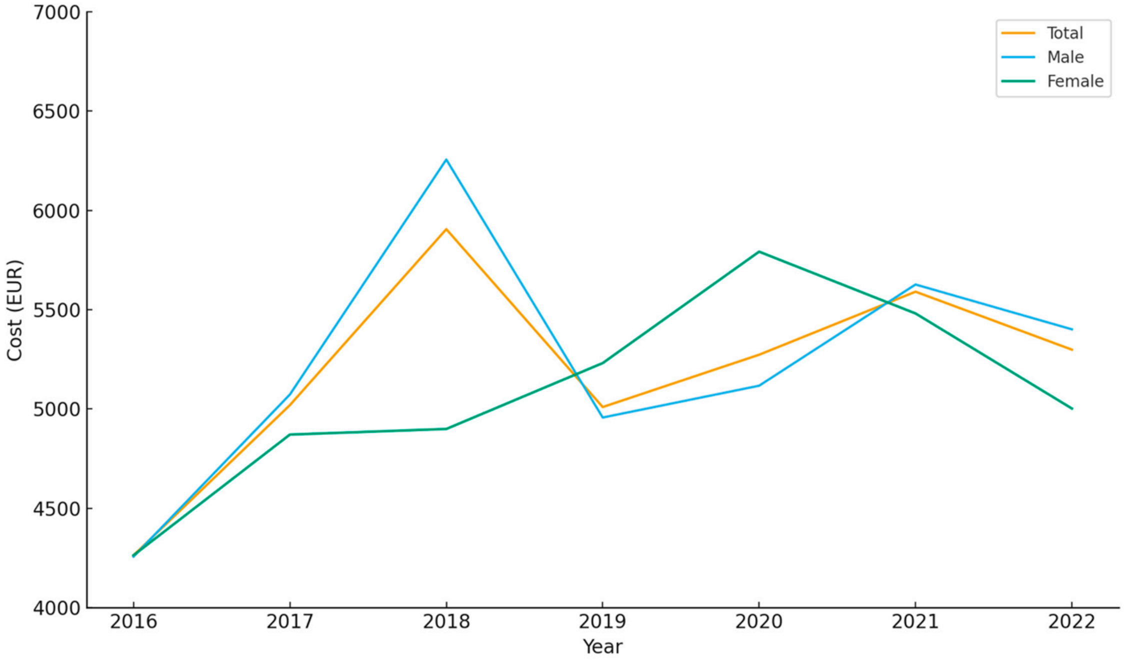

Supplementary Figure S8. Distribution of hospital costs for Wernicke encephalopathy in Spain (2016–2022).

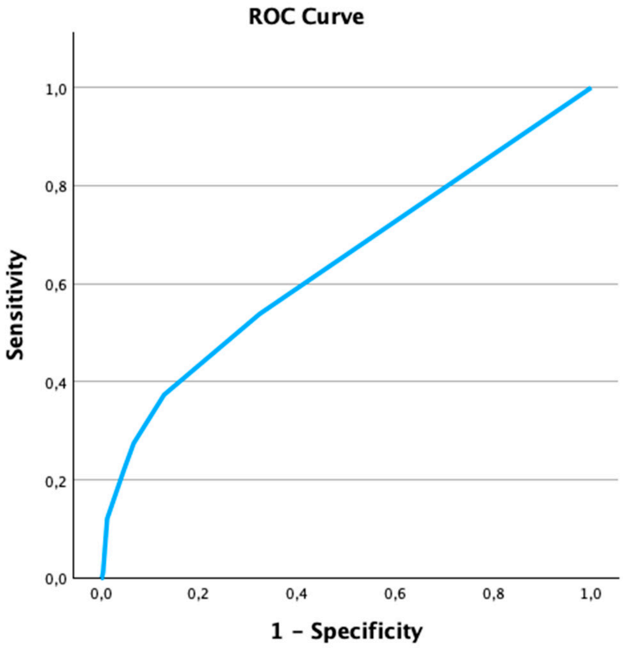

Supplementary Figure S9. ROC curve for Model 1 (clinical comorbidities).
Receiver operating characteristic (ROC) curve illustrating the discriminatory performance of Model 1, which includes the main clinical comorbidities significantly associated with in-hospital mortality, adjusted for age and sex.

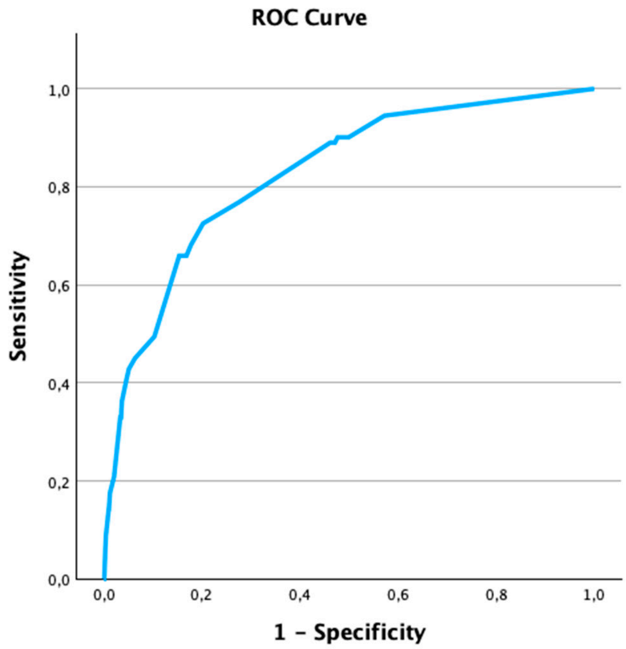

Supplementary Figure S10. ROC curve for Model 2 (clinical comorbidities and Risk of Mortality).

ROC curve showing the discriminatory ability of Model 2, which combines clinical comorbidities with the APR–DRG Risk of Mortality (ROM) index to predict in-hospital mortality.

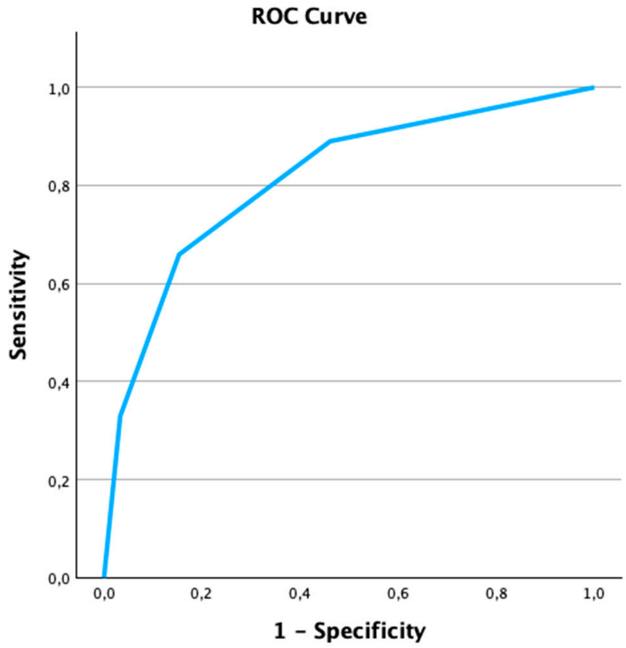

Supplementary Figure S11. ROC curve for Model 3 (Risk of Mortality only).

ROC curve depicting the predictive performance of Model 3, which includes only the APR–DRG Risk of Mortality (ROM) index as a predictor of in-hospital mortality.
